# 2,5,11,14-Tetra­oxa-8-aza­dispiro­[13.4.0]nona­deca-15,17,19-triene

**DOI:** 10.1107/S1600536810013929

**Published:** 2010-04-21

**Authors:** Quanying Gan, Liping Yang, Peng Guo, Jin Yang, Zhongyu Duan

**Affiliations:** a Hebei University of Technology, Tianjin 300130, People’s Republic of China

## Abstract

The title compound, C_14_H_21_NO_4_, has been synthesized from *o*-dihydroxy­benzene by a three-step reaction. There are two chemically equal but crystallographically independent mol­ecules in the asymmetric unit. The crystal packing is governed by C—H⋯O hydrogen bonds and C—H⋯π inter­actions, forming an infinite network.

## Related literature

For general background to crown ethers, see: Gokel *et al.* (2004 ▶); Wainwright (1997 ▶). For the synthesis, see: Lu & Wu (1989 ▶). For bond-length data, see: Allen *et al.* (1987 ▶).
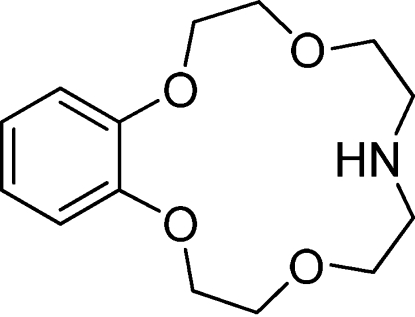


## Experimental

### 

#### Crystal data

C_14_H_21_NO_4_
*M* *_r_* = 267.32
Monoclinic, 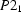

*a* = 10.771 (7) Å
*b* = 8.662 (5) Å
*c* = 15.961 (10) Å
β = 105.417 (11)°
*V* = 1435.6 (15) Å^3^
*Z* = 4
Mo *K*α radiation
μ = 0.09 mm^−1^
*T* = 294 K
0.20 × 0.16 × 0.14 mm

#### Data collection

Bruker SMART APEX CCD area-detector diffractometer
Absorption correction: multi-scan (*SADABS*; Sheldrick, 2004 ▶) *T*_min_ = 0.951, *T*_max_ = 0.987
7411 measured reflections
2717 independent reflections
1848 reflections with *I* > 2σ(*I*)
*R*_int_ = 0.043

#### Refinement

*R*[*F* ^2^ > 2σ(*F* ^2^)] = 0.065
*wR*(*F* ^2^) = 0.175
*S* = 1.05
2717 reflections
343 parameters
1 restraint
H-atom parameters constrained
Δρ_max_ = 0.43 e Å^−3^
Δρ_min_ = −0.20 e Å^−3^

### 

Data collection: *SMART* (Bruker, 2001 ▶); cell refinement: *SAINT* (Bruker, 2001 ▶); data reduction: *SAINT*; program(s) used to solve structure: *SHELXTL* (Sheldrick, 2008 ▶); program(s) used to refine structure: *SHELXTL*; molecular graphics: *SHELXTL*; software used to prepare material for publication: *SHELXTL* and local programs.

## Supplementary Material

Crystal structure: contains datablocks global, I. DOI: 10.1107/S1600536810013929/fk2017sup1.cif

Structure factors: contains datablocks I. DOI: 10.1107/S1600536810013929/fk2017Isup2.hkl

Additional supplementary materials:  crystallographic information; 3D view; checkCIF report

## Figures and Tables

**Table 1:** Hydrogen-bond geometry (Å, °) *Cg*1 is the centroid of the C15–C20 ring.

*D*—H⋯*A*	*D*—H	H⋯*A*	*D*⋯*A*	*D*—H⋯*A*
C21—H21*B*⋯O3^i^	0.97	2.56	3.511 (2)	168
C26—H26*A*⋯*Cg*1	0.97	2.69	3.646 (2)	168

Symmetry code: (i) 
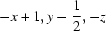
.

**Fig. 1.**
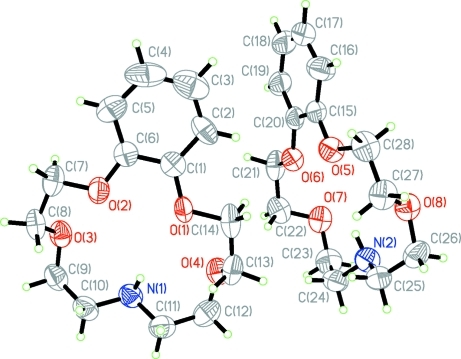
The molecular structure of (I), with atom labels and 50% probability displacement ellipsoids for non-H atoms.

**Fig. 2.**
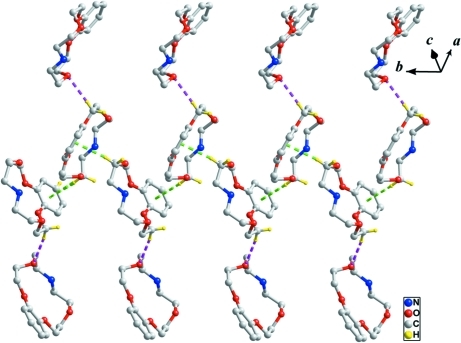
Packing diagram for (I), with H bonds drawn as dashed lines.

## supplementary crystallographic information

### Comment

Crown ethers can be subjected to diverse modifications to give a wide variety of derivatives, which could not only extend the original molecular binding ability, but also alter the molecular selectivity. Therefore, they are currently significant topics in supramolecular chemistry (Gokel *et al.*, 2004). Among the large number of compounds, aza-crown ethers are the important component (Wainwright,1997). Although the synthesis of the title compound has been reported previously (Lu & Wu, 1989), the crystal structure had not been determined. The compound is an important precursor in the synthesis of aza-crown ethers.

In the title molecule (Fig. 1), bond lengths and angles are within normal ranges (Allen *et al.*, 1987). There are two chemically equal but crystallographically independent molecules per asymmetric unit.

As shown in Fig. 2, molecules are linked by C—H···π and C—H···O hydrogen bonds to compose the crystal packing. The molecules (that contains N2) form 1D supramolecular chain by the C26—H26A···π interactions involving the C15—C20 benzene ring (centroid *Cg*1 ).In addition, through C(21)—H(21B)···O(3) hydrogen bond, the 1D supramolecular chain is connected with the molecules (that contains N1) to form an infinite network (Table 1) .

### Experimental

The title compound was prepared according the previous literature. The product was isolated, recrystallized from ethyl acetate, and then dried in vacuum to give the pure title compound in 63% yield. The single crystals suitable for X-ray analysis were obtained as colourless blocks by slow evaporation of an ethyl acetate solution.

### Refinement

Due to insignificant anomalous dispersion effects, Friedel pairs were merged before refinement.

The H atoms were included in calculated positions and refined using a riding model approximation. Constrained C—H and N—H bond lengths and isotropic U parameters: 0.93 Å and U_iso_(H) = 1.2U_eq_(C) for Csp^2^—H; 0.97 Å and U_iso_(H) = 1.2U_eq_(C) for methylene C—H; 0.89 Å and U_iso_(H) = 1.2U_eq_(N) for imino N—H.

### Crystal data

**Table:**

C_14_H_21_NO_4_	*F*(000) = 576
*M**_r_* = 267.32	*D*_x_ = 1.237 Mg m^−^^3^
Monoclinic, *P*2_1_	Mo *K*α radiation, λ = 0.71073 Å
Hall symbol: P 2yb	Cell parameters from 936 reflections
*a* = 10.771 (7) Å	θ = 2.6–22.4°
*b* = 8.662 (5) Å	µ = 0.09 mm^−^^1^
*c* = 15.961 (10) Å	*T* = 294 K
β = 105.417 (11)°	Block, colourless
*V* = 1435.6 (15) Å^3^	0.20 × 0.16 × 0.14 mm
*Z* = 4	

### Data collection

**Table:**

Bruker SMART APEX CCD area-detector diffractometer	2717 independent reflections
Radiation source: fine-focus sealed tube	1848 reflections with *I* > 2σ(*I*)
graphite	*R*_int_ = 0.043
φ and ω scans	θ_max_ = 25.0°, θ_min_ = 1.3°
Absorption correction: multi-scan (*SADABS*; Sheldrick, 2004)	*h* = −12→9
*T*_min_ = 0.951, *T*_max_ = 0.987	*k* = −9→10
7411 measured reflections	*l* = −18→18

### Refinement

**Table:**

Refinement on *F*^2^	Primary atom site location: structure-invariant direct methods
Least-squares matrix: full	Secondary atom site location: difference Fourier map
*R*[*F*^2^ > 2σ(*F*^2^)] = 0.065	Hydrogen site location: inferred from neighbouring sites
*wR*(*F*^2^) = 0.175	H-atom parameters constrained
*S* = 1.05	*w* = 1/[σ^2^(*F*_o_^2^) + (0.1029*P*)^2^ + 0.0778*P*] where *P* = (*F*_o_^2^ + 2*F*_c_^2^)/3
2717 reflections	(Δ/σ)_max_ < 0.001
343 parameters	Δρ_max_ = 0.43 e Å^−^^3^
1 restraint	Δρ_min_ = −0.19 e Å^−^^3^

### Special details

**Table:**

Geometry. All esds (except the esd in the dihedral angle between two l.s. planes) are estimated using the full covariance matrix. The cell esds are taken into account individually in the estimation of esds in distances, angles and torsion angles; correlations between esds in cell parameters are only used when they are defined by crystal symmetry. An approximate (isotropic) treatment of cell esds is used for estimating esds involving l.s. planes.
Refinement. Refinement of *F*^2^ against ALL reflections. The weighted *R*-factor wR and goodness of fit *S* are based on *F*^2^, conventional *R*-factors *R* are based on *F*, with *F* set to zero for negative *F*^2^. The threshold expression of *F*^2^ > 2σ(*F*^2^) is used only for calculating *R*-factors(gt) etc. and is not relevant to the choice of reflections for refinement. *R*-factors based on *F*^2^ are statistically about twice as large as those based on *F*, and *R*- factors based on ALL data will be even larger.

### Fractional atomic coordinates and isotropic or equivalent isotropic displacement parameters (Å^2^)

**Table:**

	*x*	*y*	*z*	*U*_iso_*/*U*_eq_	
N1	0.3503 (4)	0.5129 (6)	0.1371 (3)	0.0573 (12)	
H1	0.4359	0.5187	0.1535	0.069*	
N2	0.7083 (4)	0.0166 (6)	0.5069 (3)	0.0595 (13)	
H2A	0.7519	0.0117	0.4668	0.071*	
O1	0.7230 (3)	0.4836 (4)	0.2096 (2)	0.0488 (9)	
O2	0.6683 (4)	0.6169 (4)	0.0602 (2)	0.0579 (10)	
O3	0.4078 (4)	0.5783 (5)	−0.0279 (2)	0.0585 (11)	
O4	0.5273 (4)	0.3439 (5)	0.2723 (3)	0.0604 (11)	
O5	0.9938 (3)	0.1199 (4)	0.3925 (2)	0.0545 (10)	
O6	0.8056 (3)	−0.0162 (4)	0.2862 (2)	0.0479 (9)	
O7	0.6115 (4)	−0.1540 (5)	0.3498 (3)	0.0637 (11)	
O8	0.9812 (4)	0.0834 (5)	0.5652 (3)	0.0596 (11)	
C1	0.8137 (5)	0.4670 (6)	0.1632 (3)	0.0488 (13)	
C2	0.9266 (5)	0.3848 (7)	0.1912 (4)	0.0650 (18)	
H2	0.9467	0.3381	0.2457	0.078*	
C3	1.0097 (6)	0.3708 (11)	0.1397 (6)	0.091 (3)	
H3	1.0850	0.3136	0.1586	0.109*	
C4	0.9809 (6)	0.4413 (12)	0.0610 (6)	0.091 (3)	
H4	1.0376	0.4315	0.0265	0.109*	
C5	0.8682 (6)	0.5286 (8)	0.0300 (4)	0.0700 (18)	
H5	0.8513	0.5778	−0.0236	0.084*	
C6	0.7824 (5)	0.5399 (6)	0.0812 (3)	0.0480 (13)	
C7	0.6160 (6)	0.6595 (8)	−0.0305 (4)	0.0656 (17)	
H7A	0.6173	0.5720	−0.0681	0.079*	
H7B	0.6656	0.7429	−0.0462	0.079*	
C8	0.4788 (6)	0.7108 (7)	−0.0382 (4)	0.0621 (17)	
H8A	0.4770	0.7864	0.0063	0.075*	
H8B	0.4420	0.7573	−0.0947	0.075*	
C9	0.2861 (5)	0.6140 (8)	−0.0110 (4)	0.0655 (17)	
H9A	0.2277	0.5281	−0.0299	0.079*	
H9B	0.2492	0.7037	−0.0450	0.079*	
C10	0.2971 (6)	0.6457 (8)	0.0824 (4)	0.0623 (16)	
H10A	0.3523	0.7346	0.1009	0.075*	
H10B	0.2127	0.6701	0.0896	0.075*	
C11	0.3550 (6)	0.5332 (8)	0.2280 (4)	0.0634 (16)	
H11A	0.2695	0.5575	0.2333	0.076*	
H11B	0.4113	0.6190	0.2516	0.076*	
C12	0.4036 (6)	0.3892 (8)	0.2795 (4)	0.0719 (18)	
H12A	0.4080	0.4073	0.3401	0.086*	
H12B	0.3431	0.3058	0.2590	0.086*	
C13	0.6295 (6)	0.4306 (8)	0.3246 (4)	0.0628 (16)	
H13A	0.6468	0.3978	0.3848	0.075*	
H13B	0.6074	0.5394	0.3214	0.075*	
C14	0.7460 (6)	0.4043 (7)	0.2915 (4)	0.0636 (17)	
H14A	0.8225	0.4449	0.3323	0.076*	
H14B	0.7583	0.2949	0.2838	0.076*	
C15	1.0249 (5)	0.0476 (6)	0.3258 (3)	0.0465 (13)	
C16	1.1469 (6)	0.0433 (9)	0.3128 (5)	0.0717 (18)	
H16	1.2155	0.0918	0.3517	0.086*	
C17	1.1656 (7)	−0.0350 (12)	0.2405 (5)	0.093 (3)	
H17	1.2469	−0.0365	0.2306	0.111*	
C18	1.0679 (9)	−0.1073 (10)	0.1856 (5)	0.092 (3)	
H18	1.0827	−0.1593	0.1383	0.111*	
C19	0.9450 (6)	−0.1071 (8)	0.1971 (4)	0.0664 (18)	
H19	0.8787	−0.1600	0.1586	0.080*	
C20	0.9218 (5)	−0.0267 (6)	0.2672 (3)	0.0490 (13)	
C21	0.6986 (6)	−0.0989 (7)	0.2322 (4)	0.0611 (17)	
H21A	0.7184	−0.2081	0.2316	0.073*	
H21B	0.6784	−0.0604	0.1730	0.073*	
C22	0.5881 (5)	−0.0732 (8)	0.2704 (4)	0.0624 (16)	
H22A	0.5787	0.0362	0.2803	0.075*	
H22B	0.5090	−0.1099	0.2307	0.075*	
C23	0.5317 (6)	−0.1077 (9)	0.4040 (5)	0.0733 (19)	
H23A	0.5265	−0.1918	0.4430	0.088*	
H23B	0.4454	−0.0881	0.3677	0.088*	
C24	0.5793 (5)	0.0330 (9)	0.4564 (4)	0.0691 (18)	
H24A	0.5735	0.1207	0.4178	0.083*	
H24B	0.5247	0.0537	0.4946	0.083*	
C25	0.7610 (6)	0.1461 (8)	0.5623 (4)	0.0652 (17)	
H25A	0.7063	0.1674	0.6004	0.078*	
H25B	0.7604	0.2366	0.5265	0.078*	
C26	0.8935 (6)	0.1184 (9)	0.6157 (4)	0.0680 (17)	
H26A	0.9238	0.2094	0.6505	0.082*	
H26B	0.8928	0.0333	0.6551	0.082*	
C27	1.0351 (6)	0.2157 (7)	0.5353 (4)	0.0598 (16)	
H27A	1.0986	0.2630	0.5831	0.072*	
H27B	0.9681	0.2909	0.5120	0.072*	
C28	1.0969 (5)	0.1666 (8)	0.4664 (4)	0.0623 (16)	
H28A	1.1451	0.2515	0.4507	0.075*	
H28B	1.1554	0.0812	0.4867	0.075*	

### Atomic displacement parameters (Å^2^)

**Table:**

	*U*^11^	*U*^22^	*U*^33^	*U*^12^	*U*^13^	*U*^23^
N1	0.058 (3)	0.052 (3)	0.059 (3)	0.006 (2)	0.011 (2)	−0.004 (2)
N2	0.057 (3)	0.057 (3)	0.061 (3)	−0.001 (2)	0.010 (2)	−0.009 (3)
O1	0.055 (2)	0.043 (2)	0.043 (2)	0.0067 (17)	0.0039 (16)	0.0097 (17)
O2	0.072 (2)	0.055 (2)	0.045 (2)	0.010 (2)	0.0130 (18)	0.0087 (19)
O3	0.074 (2)	0.045 (2)	0.052 (2)	0.000 (2)	0.0089 (19)	−0.0005 (18)
O4	0.065 (2)	0.049 (2)	0.064 (3)	−0.002 (2)	0.012 (2)	0.004 (2)
O5	0.050 (2)	0.054 (2)	0.055 (2)	−0.0023 (18)	0.0058 (17)	−0.011 (2)
O6	0.0511 (19)	0.043 (2)	0.045 (2)	−0.0020 (17)	0.0036 (16)	−0.0105 (17)
O7	0.071 (2)	0.057 (2)	0.059 (3)	−0.011 (2)	0.010 (2)	−0.004 (2)
O8	0.068 (2)	0.053 (2)	0.055 (2)	−0.003 (2)	0.0108 (19)	−0.0064 (19)
C1	0.049 (3)	0.041 (3)	0.052 (3)	−0.006 (2)	0.005 (2)	−0.011 (3)
C2	0.041 (3)	0.063 (4)	0.080 (5)	0.004 (3)	−0.004 (3)	0.000 (3)
C3	0.048 (4)	0.105 (6)	0.109 (7)	0.000 (4)	0.003 (4)	−0.029 (5)
C4	0.050 (4)	0.122 (7)	0.105 (6)	−0.017 (4)	0.029 (4)	−0.043 (6)
C5	0.081 (4)	0.067 (4)	0.069 (4)	−0.010 (4)	0.032 (3)	−0.010 (4)
C6	0.051 (3)	0.038 (3)	0.053 (3)	−0.010 (3)	0.011 (3)	−0.006 (3)
C7	0.080 (4)	0.058 (4)	0.055 (3)	−0.016 (3)	0.011 (3)	0.013 (3)
C8	0.072 (4)	0.047 (3)	0.058 (4)	0.001 (3)	0.001 (3)	0.016 (3)
C9	0.063 (4)	0.061 (4)	0.064 (4)	0.014 (3)	0.004 (3)	0.003 (3)
C10	0.065 (3)	0.054 (4)	0.064 (4)	0.011 (3)	0.008 (3)	−0.009 (3)
C11	0.061 (3)	0.070 (4)	0.062 (4)	0.002 (3)	0.020 (3)	−0.011 (3)
C12	0.083 (4)	0.074 (5)	0.064 (4)	−0.019 (4)	0.029 (3)	−0.004 (4)
C13	0.080 (4)	0.060 (4)	0.043 (3)	−0.005 (3)	0.006 (3)	0.008 (3)
C14	0.078 (4)	0.046 (3)	0.050 (3)	−0.010 (3)	−0.014 (3)	0.011 (3)
C15	0.050 (3)	0.043 (3)	0.048 (3)	0.007 (2)	0.015 (2)	0.008 (3)
C16	0.057 (3)	0.079 (5)	0.084 (5)	0.008 (3)	0.026 (3)	0.011 (4)
C17	0.069 (4)	0.140 (8)	0.076 (5)	0.039 (5)	0.031 (4)	0.041 (5)
C18	0.119 (6)	0.105 (7)	0.067 (5)	0.056 (6)	0.050 (5)	0.020 (5)
C19	0.084 (4)	0.065 (4)	0.050 (4)	0.019 (4)	0.018 (3)	−0.003 (3)
C20	0.064 (3)	0.041 (3)	0.040 (3)	0.015 (3)	0.010 (2)	0.007 (2)
C21	0.082 (4)	0.044 (3)	0.042 (3)	−0.016 (3)	−0.010 (3)	−0.003 (3)
C22	0.047 (3)	0.064 (4)	0.064 (4)	−0.011 (3)	−0.007 (3)	−0.007 (3)
C23	0.054 (3)	0.081 (5)	0.086 (5)	−0.014 (3)	0.021 (3)	0.007 (4)
C24	0.058 (4)	0.081 (5)	0.068 (4)	0.016 (3)	0.017 (3)	−0.006 (4)
C25	0.075 (4)	0.065 (4)	0.058 (4)	0.012 (3)	0.021 (3)	−0.006 (3)
C26	0.081 (4)	0.073 (4)	0.050 (3)	−0.002 (4)	0.017 (3)	−0.010 (3)
C27	0.069 (4)	0.048 (4)	0.056 (4)	−0.015 (3)	0.005 (3)	−0.019 (3)
C28	0.046 (3)	0.062 (4)	0.071 (4)	−0.020 (3)	0.000 (3)	−0.010 (3)

### Geometric parameters (Å, °)

**Table:**

N1—C11	1.450 (7)	C10—H10B	0.9700
N1—C10	1.465 (8)	C11—C12	1.508 (10)
N1—H1	0.8900	C11—H11A	0.9700
N2—C24	1.416 (7)	C11—H11B	0.9700
N2—C25	1.448 (8)	C12—H12A	0.9700
N2—H2A	0.8900	C12—H12B	0.9700
O1—C1	1.382 (6)	C13—C14	1.503 (9)
O1—C14	1.439 (7)	C13—H13A	0.9700
O2—C6	1.360 (6)	C13—H13B	0.9700
O2—C7	1.454 (7)	C14—H14A	0.9700
O3—C8	1.413 (7)	C14—H14B	0.9700
O3—C9	1.440 (7)	C15—C16	1.384 (8)
O4—C13	1.409 (7)	C15—C20	1.403 (7)
O4—C12	1.422 (8)	C16—C17	1.398 (11)
O5—C15	1.352 (6)	C16—H16	0.9300
O5—C28	1.446 (6)	C17—C18	1.333 (11)
O6—C20	1.367 (6)	C17—H17	0.9300
O6—C21	1.434 (6)	C18—C19	1.383 (10)
O7—C22	1.410 (7)	C18—H18	0.9300
O7—C23	1.429 (8)	C19—C20	1.395 (8)
O8—C27	1.423 (8)	C19—H19	0.9300
O8—C26	1.428 (7)	C21—C22	1.492 (9)
C1—C2	1.377 (8)	C21—H21A	0.9700
C1—C6	1.412 (8)	C21—H21B	0.9700
C2—C3	1.372 (10)	C22—H22A	0.9700
C2—H2	0.9300	C22—H22B	0.9700
C3—C4	1.357 (11)	C23—C24	1.490 (10)
C3—H3	0.9300	C23—H23A	0.9700
C4—C5	1.403 (10)	C23—H23B	0.9700
C4—H4	0.9300	C24—H24A	0.9700
C5—C6	1.390 (8)	C24—H24B	0.9700
C5—H5	0.9300	C25—C26	1.474 (8)
C7—C8	1.516 (9)	C25—H25A	0.9700
C7—H7A	0.9700	C25—H25B	0.9700
C7—H7B	0.9700	C26—H26A	0.9700
C8—H8A	0.9700	C26—H26B	0.9700
C8—H8B	0.9700	C27—C28	1.490 (8)
C9—C10	1.489 (9)	C27—H27A	0.9700
C9—H9A	0.9700	C27—H27B	0.9700
C9—H9B	0.9700	C28—H28A	0.9700
C10—H10A	0.9700	C28—H28B	0.9700
			
C11—N1—C10	113.9 (5)	H13A—C13—H13B	108.5
C11—N1—H1	86.5	O1—C14—C13	106.8 (4)
C10—N1—H1	110.0	O1—C14—H14A	110.4
C24—N2—C25	115.8 (5)	C13—C14—H14A	110.4
C24—N2—H2A	102.8	O1—C14—H14B	110.4
C25—N2—H2A	106.3	C13—C14—H14B	110.4
C1—O1—C14	117.5 (4)	H14A—C14—H14B	108.6
C6—O2—C7	117.0 (4)	O5—C15—C16	125.0 (5)
C8—O3—C9	113.3 (5)	O5—C15—C20	114.8 (4)
C13—O4—C12	114.0 (5)	C16—C15—C20	120.1 (6)
C15—O5—C28	118.4 (4)	C15—C16—C17	119.2 (7)
C20—O6—C21	118.0 (4)	C15—C16—H16	120.4
C22—O7—C23	114.3 (5)	C17—C16—H16	120.4
C27—O8—C26	114.1 (5)	C18—C17—C16	120.5 (6)
C2—C1—O1	124.7 (5)	C18—C17—H17	119.7
C2—C1—C6	120.3 (5)	C16—C17—H17	119.7
O1—C1—C6	115.0 (4)	C17—C18—C19	121.9 (7)
C3—C2—C1	120.7 (7)	C17—C18—H18	119.0
C3—C2—H2	119.6	C19—C18—H18	119.0
C1—C2—H2	119.6	C18—C19—C20	119.2 (7)
C4—C3—C2	119.3 (7)	C18—C19—H19	120.4
C4—C3—H3	120.3	C20—C19—H19	120.4
C2—C3—H3	120.3	O6—C20—C19	125.5 (5)
C3—C4—C5	122.2 (7)	O6—C20—C15	115.5 (4)
C3—C4—H4	118.9	C19—C20—C15	119.0 (5)
C5—C4—H4	118.9	O6—C21—C22	106.3 (5)
C6—C5—C4	118.6 (7)	O6—C21—H21A	110.5
C6—C5—H5	120.7	C22—C21—H21A	110.5
C4—C5—H5	120.7	O6—C21—H21B	110.5
O2—C6—C5	126.1 (5)	C22—C21—H21B	110.5
O2—C6—C1	115.1 (4)	H21A—C21—H21B	108.7
C5—C6—C1	118.8 (5)	O7—C22—C21	108.8 (5)
O2—C7—C8	105.5 (5)	O7—C22—H22A	109.9
O2—C7—H7A	110.6	C21—C22—H22A	109.9
C8—C7—H7A	110.6	O7—C22—H22B	109.9
O2—C7—H7B	110.6	C21—C22—H22B	109.9
C8—C7—H7B	110.6	H22A—C22—H22B	108.3
H7A—C7—H7B	108.8	O7—C23—C24	113.4 (5)
O3—C8—C7	107.3 (5)	O7—C23—H23A	108.9
O3—C8—H8A	110.2	C24—C23—H23A	108.9
C7—C8—H8A	110.2	O7—C23—H23B	108.9
O3—C8—H8B	110.2	C24—C23—H23B	108.9
C7—C8—H8B	110.2	H23A—C23—H23B	107.7
H8A—C8—H8B	108.5	N2—C24—C23	112.1 (5)
O3—C9—C10	113.1 (5)	N2—C24—H24A	109.2
O3—C9—H9A	109.0	C23—C24—H24A	109.2
C10—C9—H9A	109.0	N2—C24—H24B	109.2
O3—C9—H9B	109.0	C23—C24—H24B	109.2
C10—C9—H9B	109.0	H24A—C24—H24B	107.9
H9A—C9—H9B	107.8	N2—C25—C26	112.9 (5)
N1—C10—C9	111.5 (5)	N2—C25—H25A	109.0
N1—C10—H10A	109.3	C26—C25—H25A	109.0
C9—C10—H10A	109.3	N2—C25—H25B	109.0
N1—C10—H10B	109.3	C26—C25—H25B	109.0
C9—C10—H10B	109.3	H25A—C25—H25B	107.8
H10A—C10—H10B	108.0	O8—C26—C25	113.1 (5)
N1—C11—C12	111.1 (5)	O8—C26—H26A	109.0
N1—C11—H11A	109.4	C25—C26—H26A	109.0
C12—C11—H11A	109.4	O8—C26—H26B	109.0
N1—C11—H11B	109.4	C25—C26—H26B	109.0
C12—C11—H11B	109.4	H26A—C26—H26B	107.8
H11A—C11—H11B	108.0	O8—C27—C28	108.7 (5)
O4—C12—C11	112.4 (5)	O8—C27—H27A	110.0
O4—C12—H12A	109.1	C28—C27—H27A	110.0
C11—C12—H12A	109.1	O8—C27—H27B	110.0
O4—C12—H12B	109.1	C28—C27—H27B	110.0
C11—C12—H12B	109.1	H27A—C27—H27B	108.3
H12A—C12—H12B	107.8	O5—C28—C27	106.5 (4)
O4—C13—C14	107.7 (5)	O5—C28—H28A	110.4
O4—C13—H13A	110.2	C27—C28—H28A	110.4
C14—C13—H13A	110.2	O5—C28—H28B	110.4
O4—C13—H13B	110.2	C27—C28—H28B	110.4
C14—C13—H13B	110.2	H28A—C28—H28B	108.6
			
C14—O1—C1—C2	−2.5 (7)	C28—O5—C15—C16	−14.8 (8)
C14—O1—C1—C6	176.5 (4)	C28—O5—C15—C20	165.2 (5)
O1—C1—C2—C3	178.2 (6)	O5—C15—C16—C17	−179.5 (6)
C6—C1—C2—C3	−0.7 (9)	C20—C15—C16—C17	0.4 (9)
C1—C2—C3—C4	1.1 (11)	C15—C16—C17—C18	−1.4 (11)
C2—C3—C4—C5	−0.1 (12)	C16—C17—C18—C19	0.6 (13)
C3—C4—C5—C6	−1.4 (11)	C17—C18—C19—C20	1.2 (11)
C7—O2—C6—C5	15.7 (8)	C21—O6—C20—C19	2.5 (8)
C7—O2—C6—C1	−164.7 (5)	C21—O6—C20—C15	−175.7 (4)
C4—C5—C6—O2	−178.6 (6)	C18—C19—C20—O6	179.8 (6)
C4—C5—C6—C1	1.8 (8)	C18—C19—C20—C15	−2.1 (9)
C2—C1—C6—O2	179.6 (5)	O5—C15—C20—O6	−0.4 (6)
O1—C1—C6—O2	0.6 (6)	C16—C15—C20—O6	179.6 (5)
C2—C1—C6—C5	−0.8 (7)	O5—C15—C20—C19	−178.7 (5)
O1—C1—C6—C5	−179.8 (5)	C16—C15—C20—C19	1.3 (8)
C6—O2—C7—C8	168.2 (5)	C20—O6—C21—C22	177.2 (5)
C9—O3—C8—C7	164.6 (4)	C23—O7—C22—C21	164.3 (5)
O2—C7—C8—O3	−69.4 (6)	O6—C21—C22—O7	−70.3 (6)
C8—O3—C9—C10	−83.9 (7)	C22—O7—C23—C24	−82.3 (6)
C11—N1—C10—C9	−176.0 (5)	C25—N2—C24—C23	−177.9 (5)
O3—C9—C10—N1	−59.7 (7)	O7—C23—C24—N2	−54.7 (8)
C10—N1—C11—C12	176.8 (5)	C24—N2—C25—C26	177.5 (5)
C13—O4—C12—C11	78.4 (6)	C27—O8—C26—C25	85.5 (7)
N1—C11—C12—O4	57.2 (7)	N2—C25—C26—O8	57.8 (8)
C12—O4—C13—C14	−163.3 (5)	C26—O8—C27—C28	−165.8 (4)
C1—O1—C14—C13	−175.5 (5)	C15—O5—C28—C27	−169.9 (5)
O4—C13—C14—O1	71.4 (6)	O8—C27—C28—O5	68.3 (6)

### Hydrogen-bond geometry (Å, °)

**Table:**

Cg1 is the centroid of the C15–C20 ring.

**Table:**

*D*—H···*A*	*D*—H	H···*A*	*D*···*A*	*D*—H···*A*
C21—H21B···O3^i^	0.97	2.56	3.511 (2)	168
C26—H26A···Cg1	0.97	2.69	3.646 (2)	168

Symmetry codes: (i) −*x*+1, *y*−1/2, −*z*.
